# Multivalent Conjugation of Antibody to Dendrimers for the Enhanced Capture and Regulation on Colon Cancer Cells

**DOI:** 10.1038/srep09445

**Published:** 2015-03-30

**Authors:** Jingjing Xie, Jichuang Wang, Hongning Chen, Weiyu Shen, Patrick J. Sinko, Haiyan Dong, Rongli Zhao, Yusheng Lu, Yewei Zhu, Lee Jia

**Affiliations:** 1 Cancer Metastasis Alert and Prevention Center, and Biopharmaceutical Photocatalysis of State Key Laboratory of Photocatalysis on Energy and Environment, College of Chemistry, Fuzhou University, Fuzhou 350002, China; 2 Rutgers, The State University of New Jersey, 160 Frelinghuysen Road, Piscataway, NJ, 08854, USA

## Abstract

Circulation tumor cells (CTCs) in the bloodstream of early-stage cancer patients carry the important information about valuable biomarkers and biological properties of primary tumor. However, detection and capture of CTCs are challenging owing to their low concentrations. Traditional technologies have the limited detection sensitivity and the low capture efficiency. We, herein, report an effective approach to specifically bind and capture colon cancer HT29 cells by using multiple Sialyl Lewis X antibodies (aSlex)-conjugated PAMAM dendrimers. The conjugation was characterized by using atom force microscope, UV and fluorescence measurements. The capturing and regulating HT29 cells by the aSlex-coated dendrimer conjugate were analyzed by microscopy and flow cytometry. The results indicated that the conjugate showed the enhanced capture of HT29 cells in a concentration-dependent manner and the maximum capture efficiency of 77.88% was obtained within 1 h-exposure. G6-5aSlex-FITC conjugate showed capture efficiency better than FITC-G6-COOH-5aSlex conjugate. G6-5aSlex-FITC conjugate could specifically capture HT29 cells even when the target HT29 cells were diluted with the interfering cells (e.g., RBCs) to a low concentration. The capture resulted in a concentration-dependent restraint of the cell activity. In conclusion, the aSlex-coated dendrimer conjugate displayed the great potential in capturing and restraining colorectal CTCs in blood.

Circulating tumor cells (CTCs)-driven cancer relapse and metastasis are the leading causes of cancer-related death worldwide^1^,^2^,^3^. Once tumor cells are shed from primary tumors or metastatic sites of early-stage cancer patients and enter the bloodstream, these “break-away” cells are called CTCs^3^,^4^,^5^,^6^,^7^. When cancer survivors are at remission, CTCs are usually in an extremely low concentration of 1 CTC per 10^6^ ~ 10^9^ non-cancerous hematopoietic cells^4^,^8^,^9^ without the capability of proliferation and invasion. Activated by hostile microenvironment, CTCs are gradually evolved as disseminated tumor cells (DTCs)^4^,^10^ and metastasis-initiating cells (MICs)^11^,^12^ which respectively mediated the hematogenous spread of cancer to distant sites and initiated the cancer metastasis. CTCs carry the important information about primary tumor and have valuable biomarkers distinct from those expressed on normal and carcinoma cell surfaces^5^,^13^,^14^. The increased numbers of CTCs in blood are closely associated with cancer metastatic progression and survival of patient^13^,^15^. Owing to the importance of CTCs as an indicator of poor prognosis, various approaches were exploited to efficiently isolate and capture CTCs from large populations of interfering cells.

Though many advances have been made, challenges to current techniques are still present. For example, immunomagnetic separation based on capture-agent-labeled magnetic bead was limited to the low capture yield^16^,^17^; microfluidics-based technologies that increase the cell-substrate contact frequency and duration made the device fabrication time-consuming and CTCs binding non-specific^18^,^19^,^20^; cell-size based filtration method that assumes CTCs larger than most hematopoietic cells easily missed CTCs that are smaller than pre-determined size threshold^21^,^22^,^23^,^24^; affinity-based surface capture in tailored microfluidic devices that relies on the coated antibody or ligand specific to target cancer cells resulted in the incomplete characterization of captured CTCs and the difficult release of CTCs from the bound surface^25^,^26^,^27^,^28^.

To circumvent these limitations, various nanotechnology-based cell detection and capture methods were developed. Because of the high surface area-to-volume ratio and excellent biological properties, nanomaterials broaden their application in cancer research especially in biomolecule detection^29^,^30^. It was reported that utilization of surface-enhanced Raman scattering (SERS) nanoparticles coated with epidermal growth factor (EGF) could successfully identify 1 to 720 CTCs in 1 ml of peripheral blood specimens from squamous cell carcinoma of the head and neck (SCCHN) patients^31^. Conjugation of antibody against human epithelial growth factor receptor 2 (HER2) to magnetic iron oxide nanoparticles was able to separate 73.6% human breast cancer cell SH-BR3 in 1 ml of fresh whole blood^32^. 3D-nanostructured silicon nanopillar (SiNP) substrates coated with epithelial-cell adhesion molecule antibody (anti-EpCAM) exhibited the improved cell capture efficiency of 45–65%^33^. When combined with a chaotic micromixer, the modified SiNP substrates enabled more than 95% recovery of cancer cells from the artificial blood samples^34^. Functionalized graphene oxide nanosheets with anti-EpCAM on a patterned gold surface isolated 73 ± 32.4% CTCs from blood samples of pancreatic, breast and lung cancer patients^35^. However, these cell-capture technologies were only restricted to monovalent conjugation of cancer-targeting agents to nanomaterials or substrate modification. Therefore, we hypothesize that multivalent conjugation of nanomaterials with targeting antibody for surface biomarker of CTCs may improve the capability of capturing CTCs in vitro and increase the possibility of application in vivo.

Dendrimers have been used as the versatile platforms owing to their excellent properties of uniformity, biocompatibility, high-branched structure and large numbers of functional ending groups. Biological molecules such as chemotherapeutic drugs^36^,^37^, DNA^38^, folic acid^36^,^37^,^39^, antibodies^40^,^41^,^42^ and MRI contrast agents^43^ were covalently linked to dendrimer surface for biological functions. However, traditional synthesis of dendrimer-antibody conjugates wasted the multivalency effect of dendrimers, which only employed the heterobifunctional cross-linkers such as sulfosuccinimidyl 3-(2-pyridyldithio)propionate (sulfo-LC-SPDP) and sulfosuccinimidyl-4-(N-maleimidomethyl)cyclohexane-1-carboxylate (sulfo-SMCC)^40^,^42^. To get the maximum potency of antibody or ligand conjugation, recently, biotinylated anti-EpCAM was introduced onto streptavidin coated-substrate surface by using N-hydroxysuccinimide (NHS)/maleimide chemistry^19^. 1-ethyl-3-(3-dimethylaminopropyl) carbodiimide (EDC) catalytic method was also used to make poly (amidoamine) (PAMAM) dendrimers capable to be conjugated with multiple anti-EpCAM for facilitated multivalent binding effect to get the enhanced breast CTC capture in artificial clinical blood samples^44^.

Some biomarkers are over-expressed on tumor surface not on normal cells, such as HER2, EpCAM, folate receptor, epidermal growth factor receptor (EGFR) and so on, which were usually used as the tumor targets. Different types of CTCs have their distinct biomarkers^45^, such as prostate-specific membrane antigen^42^,^46^ (PSMA, a hallmark of prostate carcinoma cells), and saliva acidifying louis oligosaccharides X^47^,^48^,^49^ (Sialyl Lewis X, Slex, a type II carbohydrate antigen for mediating the colorectal cancer metastatic process).

Here, we took the dual advantages of the unique property of PAMAM dendrimers owing to their multivalent conjugation effects and the specific interaction between antigen and antibody to prepare the antiSlex-coated dendrimer conjugates for exploring their capture efficiency and regulation mechanism on CTCs in vitro. The colorectal CTCs were not only captured in artificial blood samples but restrained in cell activity by the conjugates. This research provides a novel conceptual guidance for the effective prevention of cancer metastasis. Our another research highlight is that the captured cells can't possess the continued survival and proliferation ability, which is distinguished from the previous theory that the captured cells without damaging needed to be released from the bound surface by using the cell-surface cleaving proteins for further cellular characterization^33^,^35^.

## Results

### Synthesis and characterization of dendrimer derivatives

Surface modification of G6 PAMAM dendrimers was completed by reacting with succinic anhydride (SA). The modification led to the increased hydrodynamic diameter from 6 nm to 10.95 nm and the decreased surface charge from 14 mV to −18.81 mV. Once conjugation of fluorescein isothiocyanate (FITC) to partially carboxylated G6 PAMAM (PC G6) dendrimers, the particle size increased to 38.2 nm and the zeta potential became −8.89 mV. ^1^H NMR and FTIR spectra showed the difference in chemical structures of dendrimers and their derivatives. Compared with the unmodified dendrimers, the N-H bending vibration at 1655–1590 cm^−1^ became stronger with the carboxylation and the characterized sharp peak at 1384 cm^−1^ appeared when FITC molecules were attached onto dendrimers. With the particle size and electronegativity increased, the aggregation tendency became more serious in aqueous solution.

### Synthesis and characterization of FITC labeled or no labeled G6-aSlex conjugates

**Figure 1:**
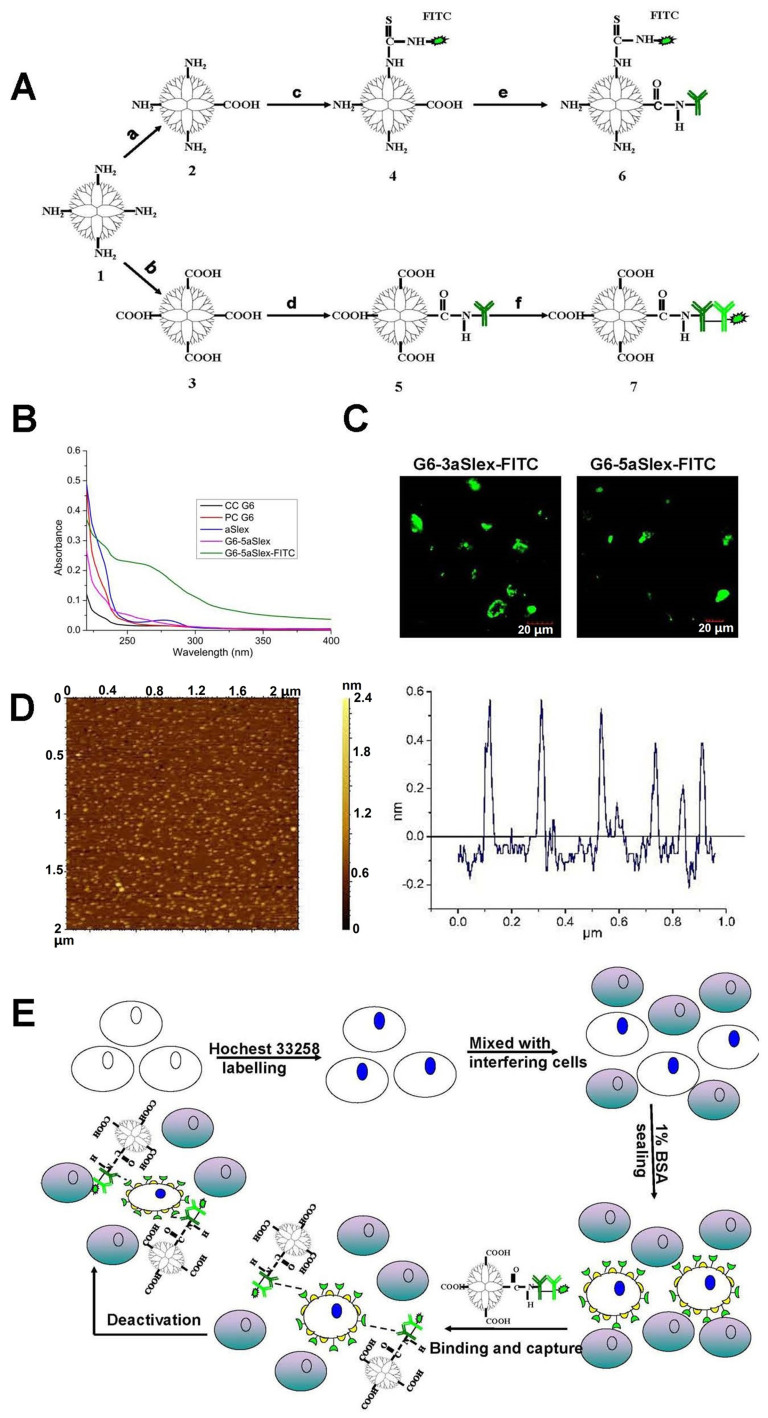
Design and characterization of conjugates using nanoscale dendrimers as scaffolds assembling multiple antibodies against carbohydrate antigen Sialyl Lewis X (aSlex) for capturing and regulating the colon cancer HT29 cells. (A), Two synthetic procedures of aSlex-conjugated dendrimers with FITC labeling by sequential conjugation of the completely carboxylated PAMAM dendrimers with FITC and aSlex (a–c–e) or by sequential conjugation of the partially carboxylated PAMAM dendrimers with aSlex and IgG/IgM-FITC (b–d–f). 1, PAMAM with amine groups; 2, PAMAM with the partially carboxylated groups; 3, PAMAM with the completely carboxylated groups; 4, PAMAM linked with FITC; 5, PAMAM conjugated with primary antibody; 6, FITC-labeled PAMAM conjugated with antibody; 7, Primary antibody-coated PAMAM conjugated with FITC-labeled secondary antibody. (B), UV/Vis spectra of dendrimers, aSlex and dendrimers-aSlex conjugates. (C), Fluorescence images of FITC labeled G6-aSlex conjugates in solution obtained by a laser confocal microscope in the channel of Alexa Fluor 488. (D), Tapping mode AFM image of G6-5aSlex conjugate (0.001% w/w) deposited on mica. The image was obtained by operating the microscope in a L state. Scan size was 2 μm. (E), Schematic view of the CTCs (HT29 cells) capture and regulation process by using aSlex-coated dendrimer conjugates.

Purified anti-Slex (aSlex) antibody and FITC labeled-second antibody (IgG/IgM-FITC) were used for the following synthesis of aSlex-coated dendrimer conjugates. If aSlex and IgG/IgM-FITC antibodies were used together, they will be abbreviated as aSlex-FITC hereafter. G6-aSlex conjugates were synthesized by conjugation of aSlex to the completely carboxylated G6 PAMAM (CC G6) dendrimers after activation with the mixture of EDC and NHS. FlTC labeled G6-aSlex conjugates were prepared either by conjugation of aSlex-FITC to CC G6 dendrimers or by conjugation of aSlex to FITC linked PC G6 dendrimers in the similar methods (Fig. 1A). The resultant conjugates with FITC labeling (former) were designated as G6-3aSlex-FITC and G6-5aSlex-FITC according to the reaction molar ratios of 1 CC G6 to 3 or 5 aSlex-FITC, and the latter were designated as FITC-G6-COOH-3aSlex and FITC-G6-COOH-5aSlex, similarly. UV spectra showed the characteristic absorption of aSlex antibody and G6-aSlex conjugates at 220 nm while PC and CC G6 dendrimers had no absorption (Fig. 1B). Green fluorescence from the FITC linked conjugates further confirmed the successful conjugation of aSlex-FITC onto the dendrimer surfaces (Fig. 1C). The functionalization of dendrimers with antibody finally resulted in the surface morphology from sphere to round pie and the particle size from 6 nm to 35 ± 5 nm (Fig. 1D). Dynamic light scattering (DLS) measurement also showed that the hydrodynamic diameter of conjugates significantly increased once aSlex was coated onto CC G6 dendrimers though the negative charge didn't change a lot. The aggregation of conjugates that were kept in phosphate-buffered saline (PBS) for long time might be ascribed to the successful surface coating of dendrimers with aSlex. However, the aggregation tendency can be reduced with the treatment of ultrasonic. The synthesized conjugates were used for the following cancer cell capture and cell activity regulation assays in vitro (Fig. 1E).

### Flow cytometric analysis of cell capture efficiency in response to various concentration or time exposures

**Figure 2:**
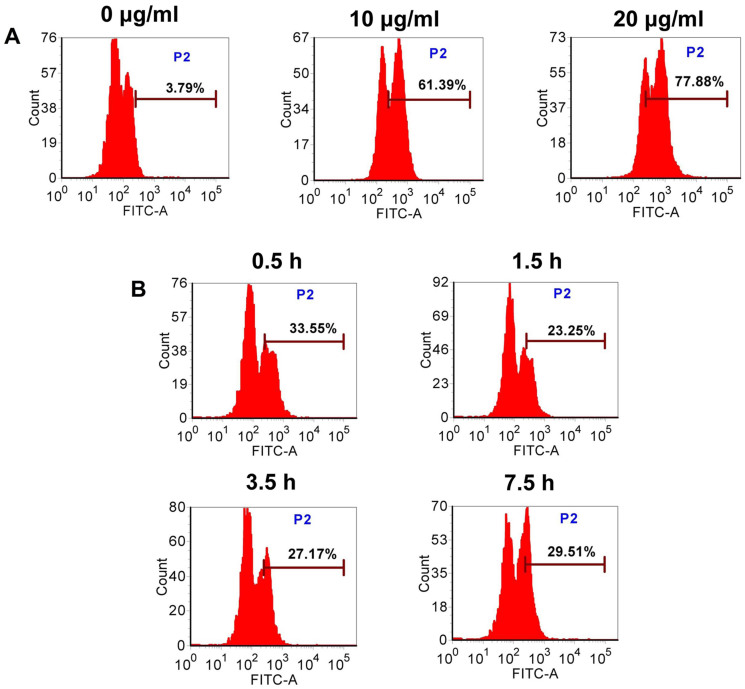
Flow cytometric analysis of G6-5aSlex-FITC conjugate capturing HT29 cells at various concentration (A) or time (B) exposures. Note: (A), the incubation time was 1 h; (B), the concentration of conjugate was 20 μg/ml. The increased percentage of FITC-positive cells indicated the enhanced capture efficiency of conjugate.

To determine the optimal incubation concentration and period for the efficient cell capture, we performed the assays in vitro. Flow cytometry was used to quantitatively analyze the capture efficiency of G6-5aSlex-FITC conjugate at different conditions. After cells exposed to the conjugate at concentration ranging from 0, 10 to 20 μg/ml, the mean capture efficiency was concentration-dependently increased from 4.83, 62.47, to 79.43% (Fig. 2A). Different time exposures indicated that the capture efficiency was nearly enhanced in a time-dependent manner from 0.5 to 1 h, and from 1.5, 3.5, to 7.5 h. However, the percentage of HT29 cells captured by the conjugate was only 29.51% at 7.5 h. It seemed that the maximum capture efficiency was obtained at 1 h rather than at 7.5 h (Fig. 2B). To make the quick and efficient-response cell capture, we chose the 1 h-incubation time and 20 μg/ml-incubation concentration as the optimal conditions for the following cell capture assays in vitro.

### Microscopic analysis for cell binding and capture behaviors

**Figure 3:**
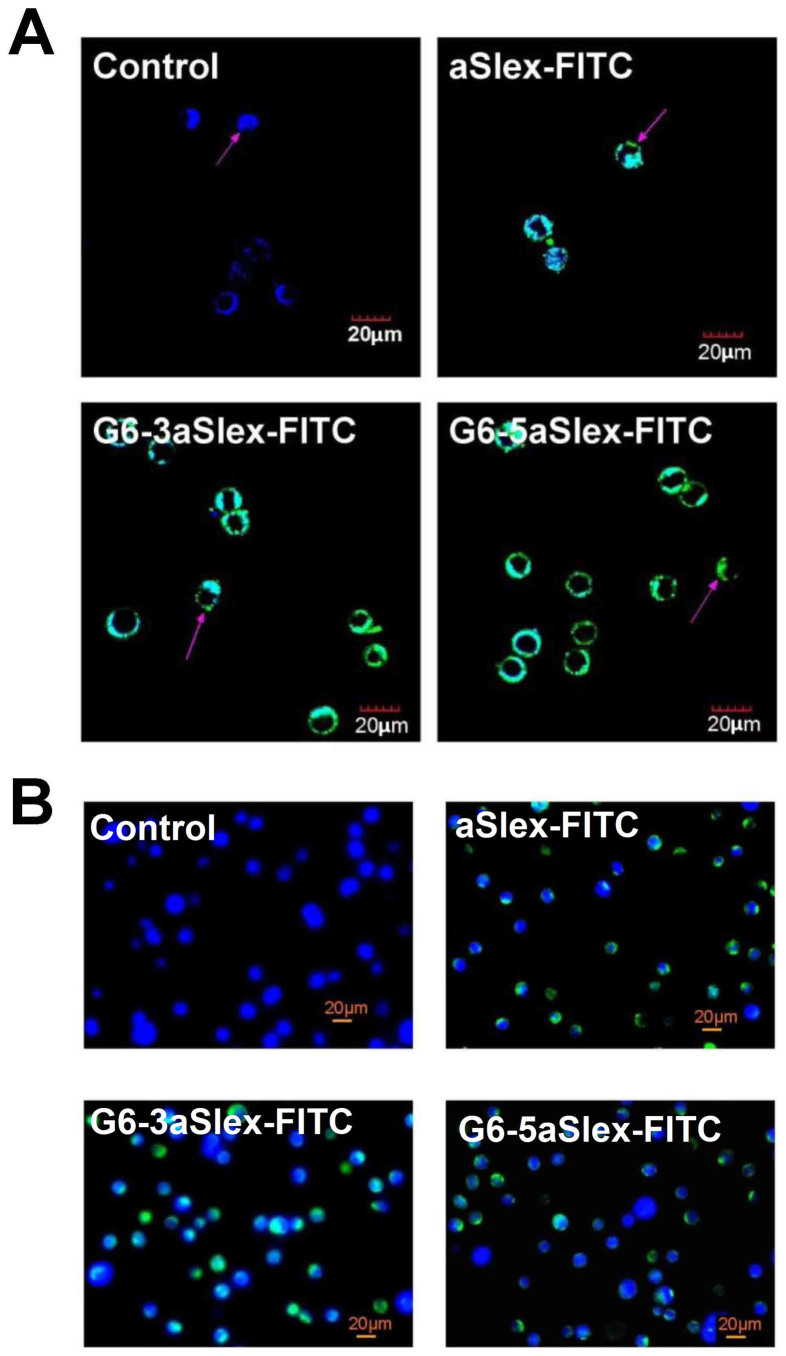
Representative images of the HT29 cells bound or captured by the FITC-labeled aSlex and G6-aSlex conjugates in one hour at 37°C. (A), Binding the adherent HT29 cells. Images were taken in the excitation channel of both Alexa Fluor 488 and DAPI. (B), Capturing the non-adherent HT29 cells. Images were taken at excitation wavelength of 488 and 365 nm. The untreated cells just showed blue color in nucleus, and the treated cells displayed blue-green merged colors in cytomembrane.

The binding affinity and specificity of aSlex-conjugated dendrimers to the adherent and non-adherent Slex-positive HT29 cells were explored. Prior to the cell binding and capture experiments, incubation of cells with 1% bovine serum albumin (BSA) excluded the non-specific interaction between HT29 cells and conjugates. Laser confocal microscope analysis was firstly carried out to the adherent cells. After 1 h of incubation with aSlex-FITC antibody or G6-aSlex-FITC conjugates, blue-green merged fluorescence was shown on the cytomembrane compared with control (blue). The increased amounts of aSlex conjugated onto dendrimer surface also assured the highly-specific interaction with target cells (Fig. 3A). To the non-adherent cells, fluorescence microscope analysis displayed that Hoechst 33258 staining just showed the presence of target cells while green color from cell membrane demonstrated the specific capture of G6-aSlex-FITC conjugates to cells (Fig. 3B). The number of bound or captured cells by conjugates was more than that by aSlex alone in any visual field. The numbers of the bound or captured HT29 cells by conjugates increased with the incubation time increased from 0.5 h to 1 h or concentration from 10 to 20 μg/ml (Figure 2). These results demonstrated that the improved capture efficiency of conjugates were mainly attributed to the increased aSlex molecules on dendrimer surface not dendrimers themselves.

### Flow cytometric analysis of cell capture efficiency in the presence of aSlex-FITC

**Figure 4:**
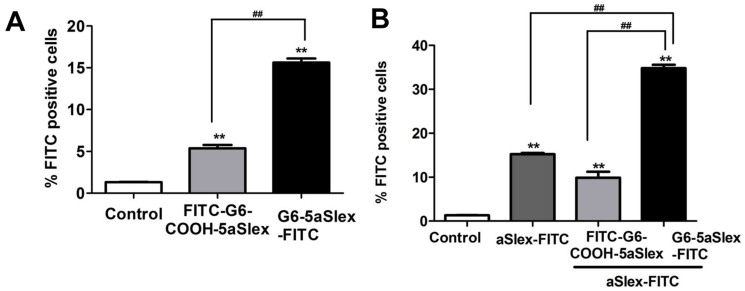
Flow cytometric analysis of the capture behaviors of G6-5aSlex-FITC and FITC-G6-COOH-5aSlex conjugates to HT29 cells in the absence or presence of aSlex. (A), Comparison of the capture efficacy between two conjugates. (B), The difference in capture efficacy between two conjugates in the presence of aSlex.

Cell-capture efficiency of conjugates to HT29 cells was evaluated by flow cytometry. The effect exerted by aSlex-FITC was investigated. After exclusion of the non-specific binding and autofluorescence both using 1% BSA and IgG-FITC isotype control, the capture efficiencies of two conjugates (G6-5aSlex-FITC and FITC-G6-COOH-5aSlex) were shown. Compared to control, the captured cells by aSlex-FITC alone demonstrated the specific antigen-antibody interaction as well as the high expression level of Slex on HT29 cell surface. G6-5aSlex-FITC conjugate had the capture efficiency up to 3-fold more than FITC-G6-COOH-5aSlex conjugate at the same exposure time of 1 h and concentration of 20 μg/ml (Fig. 4A). 30 min of pre-incubation with aSlex-FITC led to an increase in capture efficiency of 19.5% for G6-5aSlex-FITC conjugate and 4.5% for FITC-G6-COOH-5aSlex conjugate, respectively. Moreover, G6-5aSlex-FITC captured HT29 cells 24.9% more than FITC-G6-COOH-5aSlex did (Fig. 4B). The presence of aSlex-FITC significantly improved the capture efficiency of conjugates in a cooperative manner. G6-5aSlex-FITC conjugate was selected for the following experiments owing to its better capture capability than FITC-G6-COOH-5aSlex conjugate.

### Flow cytometric analysis of cell capture efficiency in the presence of interfering cells

**Figure 5:**
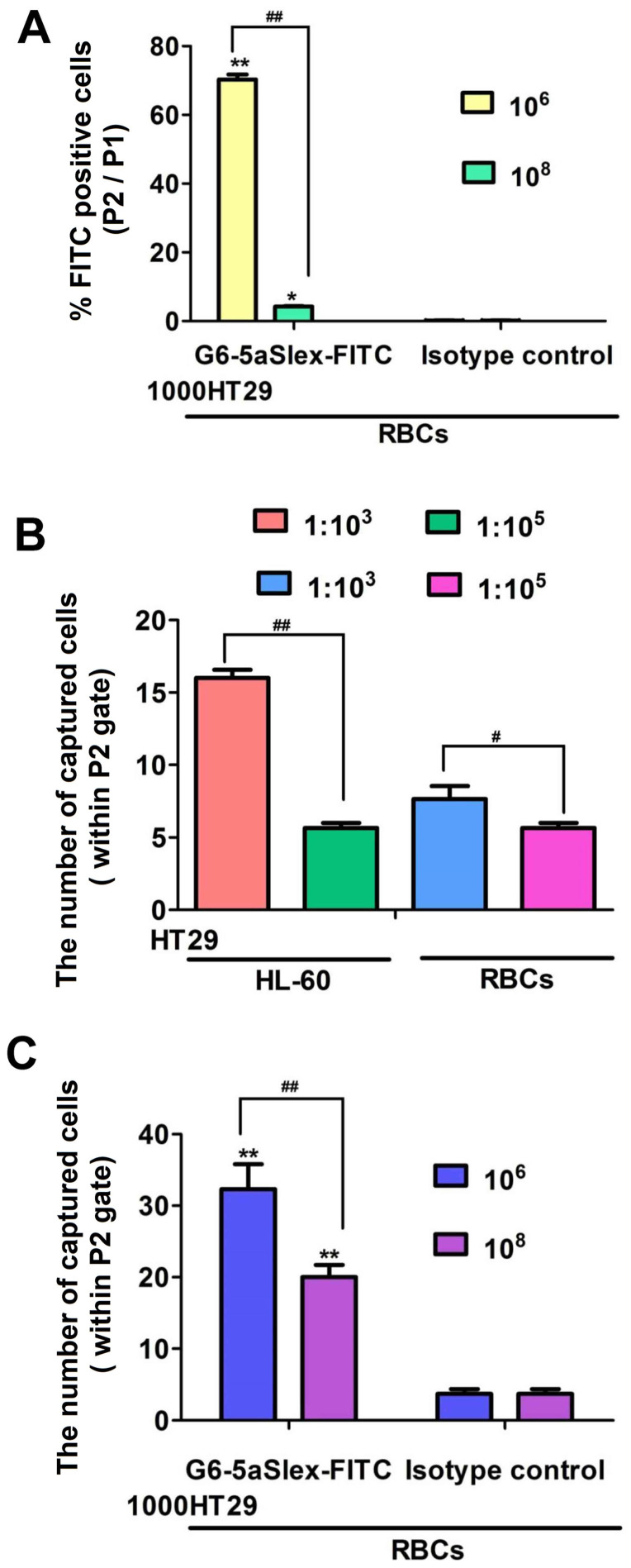
Comparison of the capture efficacy of G6-5aSlex-FITC conjugate to the target HT29 cells under the blockage from HL-60 cells or RBCs. (A), The capture efficacy was decreased when 1,000 HT29 cells were spiked with RBCs from 10^6^ to 10^8^. (B), The difference in number of HT29 cells captured by the conjugate when HT29 was mixed with HL-60 or RBC at number ratio of 1:10^3^ and 1:10^5^. (C), Comparison of the number of HT29 cells captured by the conjugate and isotype control in 1000 HT29 spiked with 10^6^ or 10^8^ RBCs.

To evaluate the efficiency of G6-5aSlex-FITC conjugate to capture CTCs, a series of artificial CTC blood samples were prepared by using HT29 cell as a colorectal CTC model and spiking HT29 cells with interfering cells at densities of 1:10^3^ or 1:10^5^ (HT29: HL-60 or red blood cells (RBCs)), or, 10^6^ or 10^8^ (RBCs) per incubation tube. After 1 h- incubation of G6-5aSlex-FITC conjugate (20 μg/ml) with the individual cell mixture, flow cytometric histogram was adjusted to show the conjugate-captured HT29 cells. The capture efficiency of conjugates was determined either by the percentage of FITC positive cells (i.e, cell number in P2 gate divided by that in P1 gate) or by the captured cell numbers within P2 gate of the flow cytometry. When 1000 HT29 cells were spiked into 10^6^ or 10^8^ RBCs, the mean % FITC positive cells sharply decreased from 70% to 4% (Fig. 5A). With the number ratio of HT29:HL-60 (RBCs) changed from 1:10^3^ to 1:10^5^, the number of captured HT29 cells also decreased (Fig. 5B). In comparison with the isotype control (3.6 ± 1.2 HT29 captured), G6-5aSlex-FITC conjugate captured more HT29 cells. However, with the spiked number of RBCs increased from 10^6^ to 10^8^, the captured cell number decreased from 32.3 ± 6.0 to 20 ± 3 in initial 1000 HT29 cells (Fig. 5C). The number of the target HT29 cells within P1 gate may be less than 100 owing to the collection of total cells in 10,000 by flow cytometry and the large numbers of spiked interfering cells. The two cell spiked experiments imitating clinical samples also showed that the obtained capture efficiency by mixing 1000 HT29 cells with 10^6^ or 10^8^ RBCs was higher than that by fixing total 10^6^ cells per incubation tube (Fig. 5B, C), which indicated the cell capture efficiency was partially correlated with the numbers of activated CTCs in blood. Meanwhile, the improved capture efficiency of aSlex-conjugated dendrimers also reflected their capture specificity to Slex-expressing HT29 cells in the presence of numerous interfering cells.

### Concentration-dependent anti-proliferation effects

**Figure 6:**
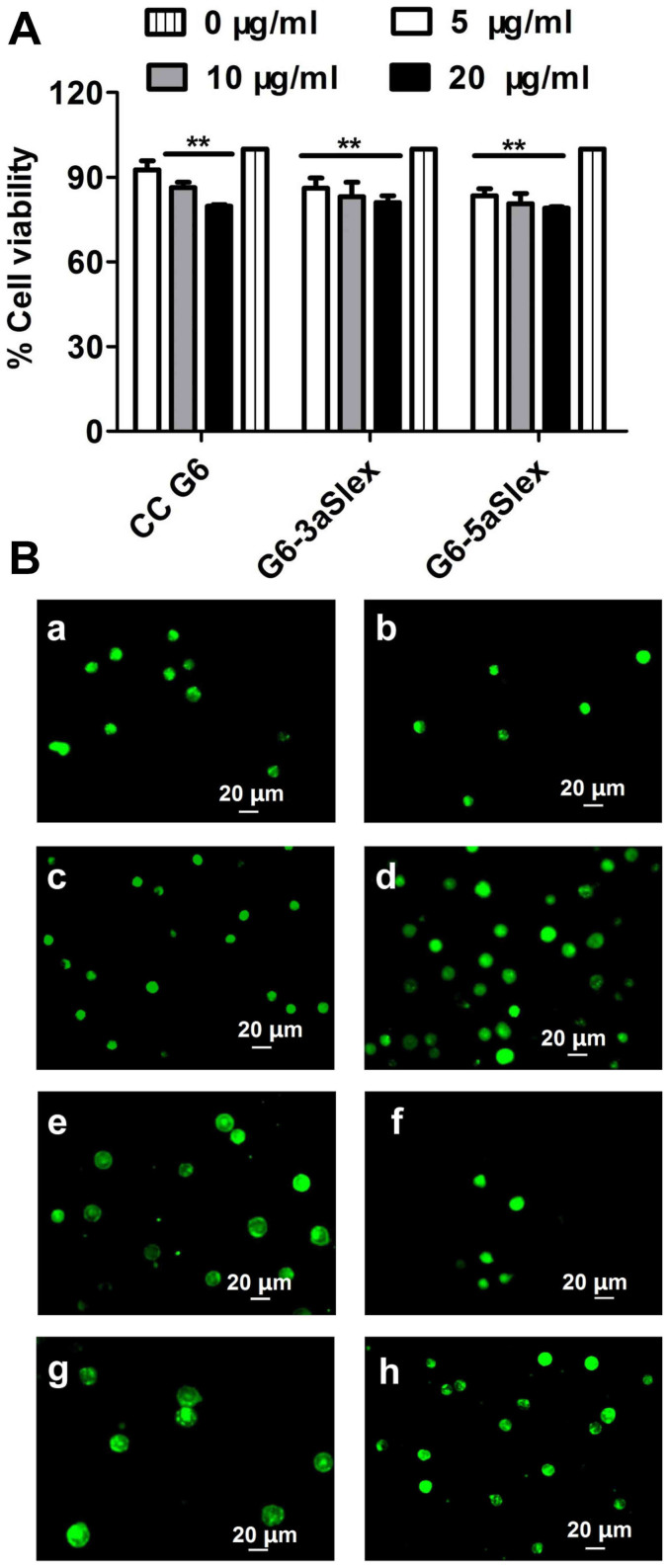
Cell growth inhibition and morphological changes induced by the CC G6 and G6-aSlex conjugates. (A), MTT assay revealed the anti-proliferation effects of conjugates. (B), AO/EB staining qualitatively showed the apoptotic status of cells. (a–b), control; (c–h), cells that were treated with CC G6 (c–d), G6-3aSlex (e–f), G6-5aSlex (g–h) at the concentrations of 10 (left) and 20 μg/ml (right), respectively.

After 48 h binding treatment with aSlex-coated dendrimers, cell viability was investigated by MTT {[3-(4,5-dimethylthiazol-2-yl)-2,5-diphenyltetrasodium bromide] tetrazolium salt} assay. The result (Fig. 6A, B) showed that both G6-3aSlex and G6-5aSlex conjugates could result in a concentration-dependent inhibition effect on HT29 cell growth with concentrations varying from 5, 10 to 20 μg/ml. However, the cell viability was decreased by conjugates by less than 30% even at the maximum concentration of 20 μg/ml, which revealed the mild regulation characters of conjugates. Five-fold molars of G6-aSlex conjugates showed the similar anti-proliferation effects as CC G6 dendrimers. The carboxylated dendrimers nearly didn't affect the cell viability. The reduction in viability of captured cells may reflect the aSlex-mediated interruption to Slex on HT29 cell surface.

### Cell cycle distribution

**Figure 7:**
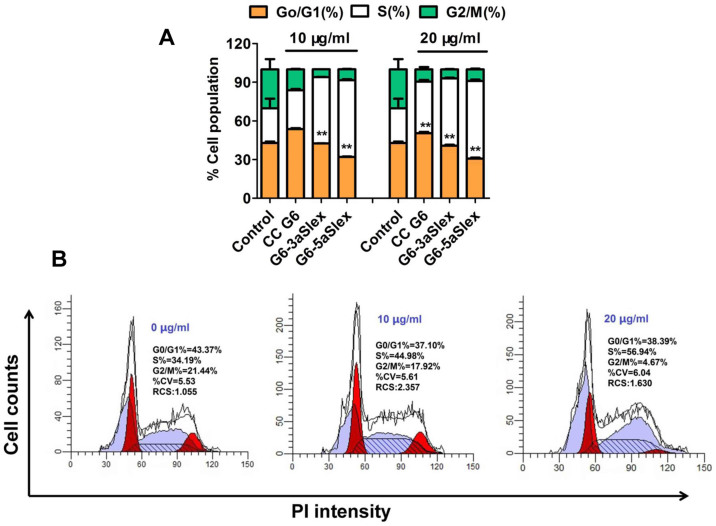
Effects of CC G6 and G6-aSlex conjugates on the cell cycle distribution. (A), Percentage of cell population in every stage (G0/G1, S, G2/M). (B), PI staining showed the cell cycle distribution induced by different concentrations of G6-5aSlex conjugate (0, 10, 20 μg/ml). In comparison with control, an obvious increase of cell population in S phase and a decrease of that in G2/M phase were found in the treated group.

To further analyze the effects of G6-aSlex conjugates on the cell cycle distribution, propidium iodide (PI) staining was used to distinguish the cell population in every phase (G0/G1, S, and G2/M). 48 h of individual incubation with G6-aSlex conjugate and CC G6 dendrimers led to a significant increase of cell population in S phase and a decrease of cell population in G2/M phase. Moreover, the percentage of cell population in S phase induced by conjugates was larger than that by CC G6 dendrimers (Fig. 7A). It seems that aSlex antibody conjugation may play an important role in the stagnation of cell cycle. With the concentration of conjugates increased from 10 to 20 μg/ml, the cell population in S phase was also concentration-dependently increased. The maximum increase of 33% in S phase by G6-5aSlex conjugate in comparison with control was consistent with the above MTT result (Fig. 7B). The inhibition of cell proliferation in S phase finally resulted in the down-regulation of captured HT29 cells without producing significant cytotoxic-killing effects.

### Qualitatively and quantitatively apoptotic analysis of HT29 cells

**Figure 8:**
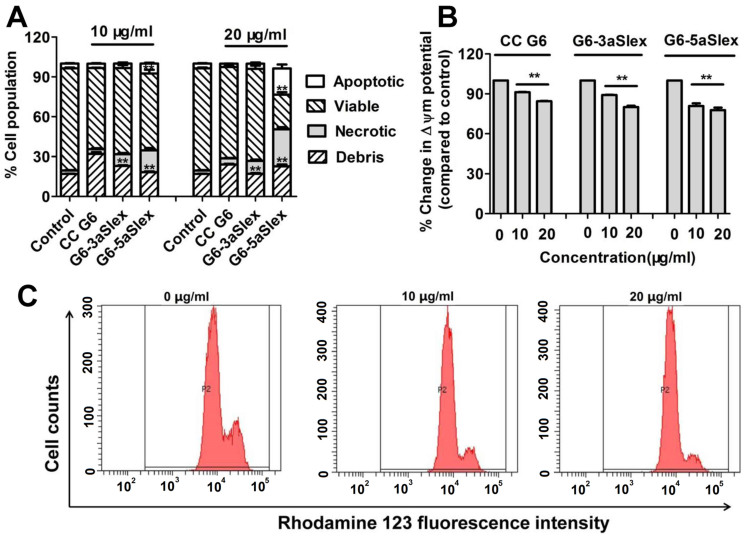
Flow cytometric analysis of apoptosis and MMP in HT29 cells induced by CC G6 and G6-aSlex conjugates. (A), Annexvin-FITC/PI staining displayed the apoptotic effects of conjugates on cells. (B), The cellular MMP was concentration-dependently decreased by conjugates. (C), Flow cytometric images showed the effects of G6-5aSlex conjugate on the cellular MMP with concentration changing from 0, 10 to 20 μg/ml.

Cell apoptosis was a crucial indicator of cell activity. How G6-aSlex conjugates affected the cell status was respectively studied by microscopic and flow cytometric analyses. The untreated cells showed clear cellular structure with the integrity of nucleolus, organelles, and plasma membrane as well as the uniform green fluorescence. CC G6 dendrimers didn't exert any effect on cell activity after 48 h-incubation. Whereas, various concentrations of G6-aSlex conjugates (0, 10, 20 μg/ml) resulted in the chromatin gathering around the ambiguous nuclear edge after exposed to cells. Significant membrane blebbing, nuclear fragmentation and apoptotic bodies were not found (Fig. 6B). Annexvin-FITC and PI staining was used to divide cells into four populations: debris, viable, apoptotic and necrotic. The apoptotic cells were only positive to FITC while the necrotic cells were positive to both FITC and PI. Flow cytometry showed that compared with control, the apoptotic and necrotic cell population concentration-dependently increased after cells incubated with G6-aSlex conjugates (especially G6-5aSlex) for 48 h (Fig. 8A). Inducing cells into early apoptotic stage might be up to the presence and numbers of aSlex conjugated onto dendrimer surface not CC G6 dendrimers.

### Changes in mitochondrial Δψm of HT29 cells

Cellular mitochondrial membrane potential (MMP) correlates with the mitochondrial function and electron transport chain activity. The untreated cells display the well-defined integral mitochondrial membrane. Cell apoptosis usually leads to the depolarization of MMP. In this assay, flow cytometric analysis was performed to show the change of MMP according to the fluorescence intensity of Rhodamine 123. Compared with control, the reduced fluorescence intensity usually indicated the decreased cellular Δψm. The ratio of the Δψm in the treated group relative to that in the control group showed that G6-aSlex conjugates reduced the cellular MMP in a modest concentration-dependent manner (Fig. 8B). When the concentration increased to 20 μg/ml, G6-5aSlex conjugate nearly resulted in 22.2% reduction in the cellular Δψm (Fig. 8C). Moreover, significant difference was found between G6-3aSlex and G6-5aSlex conjugates. It seemed that with the number of aSlex conjugated to dendrimer surface increased, the cellular MMP was more significantly deceased. Therefore, aSlex played an important role in cell activity regulation. The few influences (less than 30%) on cellular MMP produced by the aSlex coated-dendrimers were also in agreement with the above cell apoptotic analysis. These studies on regulation mechanism collectively confirmed that multivalent conjugation of aSlex to dendrimers could slightly restrain the captured CTCs.

## Discussion

Nanoscale PAMAM dendrimers mediated by multivalent binding effect^44^,^50^ were assembled with multiple aSlex antibodies. The aSlex-conjugated dendrimers as one entity were synthesized as we described previously^51^ and demonstrated by UV, fluorescence and atomic force microscope (AFM) analyses (Fig. 1A–D). The resultant FITC-labeled G6-aSlex conjugates had the high-affinity molecular recognition and binding ability in biological processes (Fig. 1E). The maximum capture efficiency was obtained after HT29 cells were exposed to the G6-5aSlex-FITC conjugate at 20 μg/ml for 1 h (Fig. 2). The reason why cell capture wasn't in a time-dependent manner may be explained in the following aspects: the capability of conjugates in binding HT29 cells was saturated at 1 h through the antigen-antibody interactions in cellular components, the capability of cellular uptake was saturated at 1 h^40^,^42^,^52^, the activity of captured cells was decreased by conjugates^51^, or the conjugate was degraded by the target cells. Within 1-h incubation, the adherent and non-adherent HT29 cells were more specifically bound and captured by G6-5aSlex-FITC conjugate (20 μg/ml) in comparison with the control and single antibody (aSlex-FITC) (Fig. 3). G6-5aSlex-FITC conjugate showed the higher capture efficiency than FITC-G6-COOH-5aSlex conjugate with or without the pretreatment of aSlex-FITC (Fig. 4). The difference in capture capability of the two conjugates might be explained from the two aspects: large amounts of functional carboxyl end groups of dendrimers made it accessible to assemble with multiple antibodies; and the higher efficiency of fluorescence labeling for antibody than that for dendrimers increased the signal intensity and improved the detection probability. To further determine the capture specificity and capture efficiency of G6-5aSlex-FITC conjugate in the presence of large concentrations of interfering cells (HL-60 and RBCs), the related capture assays were carried out. The % FITC positive cells or the captured cell number was decreased with the cell mixture ratio changed from 1:10^3^ to 1:10^5^ or the number of spiked interfering cells increased from 10^6^ to 10^8^ (Fig. 5). It seemed that the presence of large populations of HL-60 cells and RBCs hindered the specific recognition and capture of conjugate to the target HT29 cells. The low capture efficiency was attributed to the huge blockage from interfering cells and the reduced interaction chance between conjugate and the target cells^33^. The interference effect of RBCs seemed to be stronger than that of HL-60 cells, which might be up to the different cell features and types. Cell anti-proliferation, cell cycle stagnation, cell apoptosis and cellular MMP analyses consistently revealed the mild regulation properties of the aSlex-coated G6 PAMAM dendrimer conjugates that led to the restraint of cell activity without producing a significant cytotoxic-killing effect (Figs. 6,7,8). It seemed that the down-regulation on HT29 cells might be mainly ascribed to the interruption of multiple aSlex antibodies on dendrimer surface to Slex on HT29 cell surface. The above results collectively demonstrated that aSlex conjugated-dendrimers could specifically recognize and capture the Slex-expressing HT29 cells not RBCs and multiple conjugation of aSlex to dendrimer surfaces largely contributed to the improved capture efficiency and the enhanced down-regulation of cell activity in comparison with the effect of monovalent conjugates.

In conclusion, a cell-capture platform that utilizes nanostructured dendrimers as scaffolds to assemble multiple antibodies was firstly exploited for colorectal CTCs model capture in vitro. A series of capture assays collectively demonstrated the specific binding and capture of dendrimer-aSlex conjugates to HT29 cells after 1-h incubation in the absence or presence of abundant interfering cells (HL-60 cells and RBCs). The enhanced local topographic interactions between dendrimer-aSlex surface and nanoscale receptors on the cell surface as well as the multiple aSlex/Slex interactions collectively contributed to the efficient and specific cell capture. Importantly, the study on regulation mechanism, for the first time, showed that the viability of captured CTCs with this platform was decreased in a modest concentration-dependent manner. The increased cell capture and regulation capability of conjugates enabled the design of novel functionalized nanomaterials to capture and restrain CTCs in blood.

## Methods

### Materials

PAMAM dendrimers (Generation 6, theoretical MW 624,00 Da, ethylenediamine core) were purchased from Shandong Weihai Chenyuan New Silicone Materials, Co. Ltd. SA, Deuterium Oxide (99.9 atom % D, D_2_O), EDC, and NHS were obtained from Aladdin Reagent Co., Ltd. FITC, MTT, PI, Rhodamine 123, 4,6-diamino-2-phenyl indole (DAPI) and BSA (fraction V) were purchased from Sigma-Aldrich. The cell cycle and apoptosis detection kits, and antibodies such as aSlex, IgG/IgM-FITC, IgG-FITC, and APC labeled anti-CD45 that were used for flow cytometric analysis were provided by BD company. All other chemicals, unless otherwise specified, were all purchased from Sinopharm Chemical Reagent Co., Ltd and used without further purification. The blood samples of healthy human or SD rat were provided by Fuzhou General Hospital of Nanjing Military Command and our laboratory, respectively.

### Preparation of dendrimer derivatives

G6 PAMAM dendrimer derivatives were ahead of time prepared before the synthesis of a series of conjugates. PC G6 and CC G6 dendrimers were prepared for the subsequent conjugation of FITC and antibody by dendrimers reacting with SA. Briefly, G6-(NH_2_)_256_ (80 mg, 1.28 μmol) was dissolved in 2 ml DMSO, and reacted with 32.8 mg SA (328 μmol, 1:1 molar ratio) under vigorous stirring overnight for the preparation of PC G6. For CC G6 (G6-(COOH)_256_), ten times excess molar ratio of SA (246 mg, 660 μmol) was added to the G6-(NH_2_)_256_ (60 mg) solution in 2 ml DMSO and mixed well under stirring overnight. The resultant G6 PAMAM derivatives were recovered from DMSO solution via dialysis against DDI water overnight to remove the unreacted SA as well as organic solvent, and obtained as powder by lyophilization. The synthesized PC G6 dendrimers (G6-(COOH), 24 mg) were further reacted with five times excess molar ratio of FITC (1.4 mg) in 2 ml DMSO, and 0.168 g NaHCO_3_ (1 M) was added to make the remaining amine ends (−NH_2_) of dendrimer easy to be covalently coupled with sulfur cyanide group (N = C = S^−^) of FITC under the pH value of 9.5, then the reaction was conducted for two days. The resultant FITC-conjugated dendrimers were also dialyzed in deionized distilled (DDI) water for two days, followed by lyophilization. Finally, fluorescence or non fluorescence- labeled antibodies (aSlex and aSlex-FITC) were conjugated onto these dendrimer derivatives as described in the following sections.

### Synthesis of G6-aSlex conjugates with or without FITC labeling

For G6-aSlex conjugates, CC G6 dendrimers (0.55 μg, 7.9 pmol) were dissolved in 2 ml PBS buffer, and the carboxylic ends of dendrimers were activated with the equal molar ratios of EDC (75.7 ng, 395 pmol, 50 molar excess) and NHS (45.46 ng, 395 pmol, 50 molar excess) at room temperature for 1 h. The activated dendrimers were equally divided into two vials, and reacted with aSlex at 11.85 pmol (3 molar excess) for G6-3aSlex conjugate, 19.75 pmol aSlex (5 molar excess) for G6-5aSlex conjugate, individually. For FITC labeled G6-aSlex conjugates, two different methods were employed. One was that CC G6 was pre-incubated with aSlex for 12 h, and then treated with IgG/IgM-FITC for another 12 h. The other was that aSlex was directly conjugated to FITC linked PC G6 dendrimers (G6-COOH-FITC). The synthetic procedures were similar to those described above. All the reactions were under vigorous stirring overnight in dark. Finally, all the conjugates were purified via dialysis (10,000 MWCO) against DDI water overnight followed by lyophilization.

### Characterization of dendrimer derivatives and conjugates

For G6 PAMAM dendrimer derivatives and conjugates, the chemical structures were confirmed by ^1^H NMR (an AVANCE III 500MHz NMR system, Bruker, Switzerland) in D_2_O and FTIR (a Nicolet 360 Fourier Transform IR spectrometer, Nicolet Instruments, Inc.). The surface charge (zeta potential, mV) and particle size (diameter, nm) were measured by a Zeta potential/dynamic light scattering analyzer (Zetaplus/90plus, BIC, Brookharen). The morphological properties were evaluated by an AFM 500 (Agilent Technologies) in tapping probe (40 N/m) and AC mode. The presence of FITC labeled-antibody on dendrimer surface was measured by a fluorescence inverted microscope (Axio Observer A1, Zeiss). The number of FITC molecules in each PC G6 dendrimer and the amount of aSlex molecules in each conjugate were determined by fluorescence intensity at λ_530 nm_ and UV absorption at λ_220 nm_, respectively.

### Cell culture

Human HL-60 leukemia cells were obtained from our in-house frozen cell stock cryopreserved in ampoules in a large repository and cultured in RPMI 1640 medium supplemented with heat-inactivated fetal calf serum (FCS, 10%), penicillin/streptomycin (P/S, 1%) in a humidified atmosphere of 5% CO_2_ at 37°C. Human HT29 colorectal carcinoma cells were purchased from the Type Culture Collection of the Chinese Academy of Sciences, Shanghai, China, and maintained in McCoy's 5a medium supplemented with 10% fetal bovine serum (FBS) and 1% P/S in a humidified atmosphere of 5% CO_2_ at 37°C. HT29 cells at final density 10^5^–10^6^ cells/ml were grown in 96-well plates for MTT assays, 6-well plates for flow cytometry, and 35 mm dishes with glass coverslips in the bottom for confocal microscope analysis.

### Animal experiments

All animals used in these investigations were handled in accordance with the Guide for the Care and Use of Laboratory Animals (National Research Council, 1996), and approved by the institutional animal care and use committee of Fuzhou University. All possible efforts were made to minimize the animals' suffering and to reduce the number of animals used.

### Blood samples

Blood samples were either withdrawn from healthy human after obtaining informed consent under an hospital institutional review board (IRB)-approved protocol or from the healthy SD rats in our laboratory. All specimens were collected into EDTA-containing vacutainer tubes according to the standard protocol and processed within 24 h. RBCs were separated from white blood cells (WBCs) via centrifugation with blood at 14,000 g. HT29 cells, HL-60 cells and RBCs were counted and mixed at certain number ratio for cell capture assays in vitro.

### Flow cytometry

Flow cytometric analysis was performed on a Becton Dickinson FACS Aria III cell sorter with laser excitation set at 488. PI or FITC (Rhodamine 123)–derived fluorescence signals were detected through 585 and 530 nm bandpass filters, respectively. Gating strategy was used to set P1 gate for selecting the target HT29 cells according to the forward versus side scatter histograms. In cell capture assays, side angle scattered light (SSC) versus FITC histogram showed the specific binding of G6-aSlex conjugates to HT29 cells. In cell activity regulation, SSC versus PI or Rhodamine 123 histogram was displayed for cell cycle and MMP analyses, respectively. For analysis of apoptosis in HT29 cells, compensation was made for FITC and PI dyes followed by dot plots to show the percentage of cell population within each plot quadrant. Finally, data acquisition was collected based on 10,000 cells satisfying the light scatter criteria and initial data analysis was performed using the BD FACS Diva software provided with the system.

### Concentration- or time- response cell capture

To investigate the capture capability of G6-5aSlex-FITC conjugate to the non-adherent HT29 cells in vitro in response to various concentration or time exposures, flow cytometric analysis was conducted. HT29 cells (10^6^/tube) after 30-min treatment with PBS containing 1% BSA (1% PBSA) were respectively treated in two ways: One was that cells were individually incubated with the conjugate at various concentrations (0, 10, 20 μg/ml) at 37°C water bath for 1 h. Another was that cells were exposed to the serum-free medium containing conjugate (20 μg/ml) at different time courses (0, 0.5, 1, 2, 4, 8 h). After removal of the unbound conjugate via centrifugation, cells in each tube were suspended with 500 μl PBS for multiparametric fluorescence-activated cell sorting (FACS) analysis. The capture efficiency was obtained based on the % FITC positive cells shown within P2 gate. In comparison with the control, the optimal incubation time and concentration for cell capture in vitro were finally determined.

### Cell binding and capture studies

To investigate the capability of conjugates in binding and capturing HT29 cells, microscopic observation and flow cytometric analysis were both used.

#### Microscopic analysis

The binding and capture behaviors between aSlex-FITC antibody and G6-aSlex-FITC conjugates were qualitatively compared. For the adherent cells, cells at the density of 10^5^ cells/ml were pre-inoculated on 35 mm dishes with glass coverslips in the bottom, followed by treated with 1% PBSA to exclude the autofluorescence and non-specific binding, then individually exposed to aSlex-FITC alone or G6-3aSlex-FITC and G6-5aSlex-FITC conjugates (20 μg/ml) for 1 h at 37°C in dark. Cells were washed and fixed with nuclei stain DAPI solution (10 μg/ml, blue color) for another 15 min. After cells were rinsed and covered with serum-free medium, an Olympus FluoView 1000 laser scanning confocal microscope was used to take the images of the bound cells.

For the non-adherent cells, HT29 cells (10^6^/tube) were firstly stained with Hoechst 33258 (blue color) for 15 min indicating the presence of cell nucleus, then mixed with fluorescence-labeled antibody (aSlex-FITC) and conjugates (G6-3aSlex-FITC and G6-5aSlex-FITC) for 1 h at 37°C water bath in dark after treatment with 1% PBSA for 30 min. After removal of the unbound conjugates, the captured cells were taken images by a fluorescence inverted microscope (Axio Observer A1, Zeiss, Germany).

#### Flow cytometric analysis

The capture capability of conjugates on the non-adherent cells was quantitatively determined by flow cytometric analysis. Immunoglobulin labeled with the same fluorescein (IgG-FITC) was used as the isotype control to normalize the fluorescence intensity. P1 gate was set to determine the target HT29 cells and P2 gate was set to show the captured cells by conjugates. The percentage of FITC positive cells (i.e., % FITC positive cells) was calculated based on the number of the target cells captured (P2) divided by that of the target cells initially selected (P1). The capture behaviors of conjugates under various conditions were individually studied.

The specific receptor-mediated binding experiment was carried out by using the free aSlex-FITC antibody prior to the G6-5aSlex-FITC conjugate. Whether the presence of aSlex-FITC will block or accelerate the capture of conjugates to Slex-expressing HT29 cells was validated by us. HT29 cells (10^6^/tube) were firstly treated with 1% PBSA for 30 min followed by incubated with aSlex-FITC for 30 min, then with G6-5aSlex-FITC conjugate (20 μg/ml) for 1 h at 37°C in dark. Finally, fluorescence intensities from the conjugate-bound cell surfaces were quantified on a Becton Dickinson FACS Aria III analyzer.

The capture capability of conjugates on the target HT29 cells in the presence of interfering cells was also explored by us. HT29 cells were counted and spiked with different numbers of HL-60 cells or RBCs of SD rat or human at a mixture ratio of 1:10^3^ and 1:10^5^. To exactly imitate the conditions of CTCs in blood, the total number in each incubation tube was controlled to be 10^6^ or the number of HT29 cells in each incubation tube was 10^3^. After co-incubation with isotype control or G6-5aSlex-FITC conjugate (20 μg/ml) for 1 h and removal of the unbound conjugates, cells were examined by the flow cytometry.

### Cell activity regulation

#### Cytotoxicity assay

The anti-proliferation effects of conjugates on HT29 cells were tested by MTT assay. HT29 cells were grew on 96-well plates in the confluence of 70%–80% and exposed to conjugates (G6-3aSlex and G6-5aSlex) at various concentrations (0, 10, 20 μg/ml). After 48 h co-incubation, 100 μl serum-free medium containing 1 mg/ml MTT solution was added to each well for 4 h. The supernatant was aspirated and 150 μl DMSO was added to dissolve the water-insoluble blue formazan. The optical density of each well was read on an ELISA reader at a wavelength of 570 nm and the survival rate was calculated by the ratio of the absorption values in the treated group compared to those in the control group.

#### Cell cycle analysis

To evaluate the effects of conjugates on the distribution of cell population in every phase (G0/G1, S, and G2/M), cell cycle analysis kit was used. Briefly, HT29 cells were inoculated on 6-well plates. After treatment with the above conjugates (0, 10, 20 μg/ml) for 48 h, cells were trypsinised and washed with ice-cold PBS for three times. Then cells were collected and fixed with 70% ice-cold ethanol overnight at −20°C. Fixed cells were washed and stained with PI solution at 37°C for 15 min. Data acquisition and analysis was finally read on a flow cytometer (BD FACS Aria III).

#### Morphological observation

HT29 cells were cultivated on the 6-well plates at the density of 5 × 10^4^ cells/ml and incubated with various concentrations of conjugates (0, 10, 20 μg/ml) for 48 h. The treated cells were trypsinized and washed with PBS. Finally, 25 μl cell suspension at the density of 1 × 10^6^/ml was stained with 1 μl mixed dye reagents (acridine orange/ethidium bromide, AO/EB) or 10 μg/ml DAPI solution in dark for 15–30 min. Images were respectively taken by a fluorescence inverted microscope (Zeiss, Axio Observer A1) at the excitation wavelength of 510 and 340 nm.

#### Apoptotic analysis

Annexin V-FITC apoptosis detection kit was used to quantitatively determine the apoptotic status of the treated HT29 cells with conjugates. Cells were firstly inoculated on the 6-well plates and incubated with conjugates (0, 10, 20 μg/ml) for 48 h followed by washed with PBS for three times, and harvested by centrifugation. Annexin V-FITC (5 μl), PI (5 μl) and binding buffer (500 μl) were mixed and added to each tube, then kept in dark for 15 min. The cells in different stages were analyzed within 1 h by flow cytometry (BD FACS Aria III).

#### MMP measurement

The changes of MMP in HT29 cells were evaluated with Rhodamine 123 staining. The assay was based on the accumulation of Rhodamine 123 in mitochondria with the cell apoptosis. Firstly, cells were cultured and treated with the above conjugates (0, 10, 20 μg/ml) for 48 h. Then cells were trypsinized and washed with PBS. Cells were collected after centrifugation and incubated with 500 μl of 10 μg/ml Rhodamine 123 working solution at 37°C for 10–30 min. The change of MMP was finally up to the Rhodamine 123-derived fluorescence intensity examined by the flow cytometry (BD FACS Aria III).

### Statistical analysis

Cell capture and cell cytotoxicity assays were individually performed at six times in parallel. Other experiments including cell cycle, cell morphology, cell apoptosis, cellular MMP analyses were conducted independently and repeated at least three times. Data were expressed as the means ± standard deviations (SD). Statistical analysis was done by Student's t-test and one-way analysis of variance (One-ANOVA). Multiple comparisons of the means were made through One-ANOVA analysis and demonstrated by the least significance difference (LSD) test (IBM SPSS Statistics 19.0). The symbol of * and ** represented the comparison between sample and control, while **^#^** and **^##^** represented the comparison between any two samples. A probability value of <0.05 was considered significant (* and **^#^**), and <0.01 was considered extremely significant (** and **^##^**).

## Author Contributions

J.X. and L.J. conceived the study. J.X. designed the experiments. J.X., H.C. and R.Z. performed most and J.W., W.S., Y.L., Y.Z. did some of the experiments. J.X., J.W., P.J.S. and H.D. analyzed and interpreted the data. L.J. and J.X. co-wrote the paper. All authors discussed the results and commented on the manuscript.

## Additional information

Ethics Statement: all methods including the human experiments were carried out in accordance with the approved guidelines.
